# XPA, XPC, and XPD Modulate Sensitivity in Gastric Cisplatin Resistance Cancer Cells

**DOI:** 10.3389/fphar.2018.01197

**Published:** 2018-10-17

**Authors:** Natalia Pajuelo-Lozano, Jone Bargiela-Iparraguirre, Gemma Dominguez, Adoracion G. Quiroga, Rosario Perona, Isabel Sanchez-Perez

**Affiliations:** ^1^Departamento de Bioquímica, Facultad de Medicina, Instituto de Investigaciones Biomédicas de Madrid, Consejo Superior de Investigaciones Científicas – Universidad Autónoma de Madrid, Madrid, Spain; ^2^Instituto de Investigaciones Biomédicas, Consejo Superior de Investigaciones Científicas – Universidad Autónoma de Madrid, Madrid, Spain; ^3^Departamento de Medicina, Facultad de Medicina, Instituto de Investigaciones Biomédicas de Madrid, Consejo Superior de Investigaciones Científicas – Universidad Autónoma de Madrid, Madrid, Spain; ^4^Departamento de Quimica Inorganica, Facultad de Ciencias, Universidad Autónoma de Madrid, Madrid, Spain; ^5^CIBER of Rare Diseases, Valencia, Spain; ^6^Unidad Asociada de Biomedicina, University of Castilla–La Mancha, Consejo Superior de Investigaciones Científicas, Albacete, Spain

**Keywords:** gastric cancer, apoptosis, cisplatin, NER repair, Bcl-2 family

## Abstract

Cisplatin is an election drug widely used in clinic for the treatment of advanced gastric cancer. However, the heterogeneity of the gastric tumors and its resistance to the drugs, make in some cases the response very low and the prognosis unpredictable. In this manuscript we aim to find the molecular processes involved in cisplatin-induced apoptosis in two gastric cancer cell lines with different sensitivity to the treatment: AGS and MKN45. The apoptosis induction is higher in MKN45 than in AGS cells in response to CDDP. The intrinsic apoptotic pathway study revealed that MKN45 cells undergo degradation of Mcl-1 together with an increase of Bid and Bad levels, which results in sensitivity to CDDP. In addition, DNA repair NER pathway is impair in MKN45 cells due to low levels of XPC and the absence of translocation of XPA and XPD to the nucleus after stimuli. Altogether, these results suggest that NER and Bcl-2 protein family proteins are potential targets to improve the response to cisplatin treatment.

## Introduction

Gastric cancer (GC) is currently the fourth most diagnosed cancer worldwide (Mandeville et al., 2009). Despite recent improvements in survival rates, there are still too many patients diagnosed at advanced stages, for which the current clinical regimen is not efficient. The standard therapy regimen consists of gastrostomy and adjuvant radio-chemotherapy with cisplatin (CDDP) and 5-Fluorouracil (5FU) treatment (Macdonald et al., 2001; Orth et al., 2014).

Radiation (IR) and chemotherapy cause a variety of DNA lesions, which in turn activate the DNA damage response (DDR) (Basu and Krishnamurthy, 2010; Orth et al., 2014). Checkpoint Kinase 1 (Chk1) and Chk2, key effectors in DDR, are multifunctional Ser/Thr kinase proteins, which represent crucial components of all cell cycle checkpoints (Bartek and Lukas, 2003). They are both involved in drug resistance and also coordinate the crosstalk between different checkpoints to ensure genome stability (Chila et al., 2013; Dillon et al., 2014; Bargiela-Iparraguirre et al., 2016). For instance, previous *in vitro* studies from our lab have demonstrated that in GC, elevated levels of Chk1 and MAD2 confer resistance to radiotherapy and sensitivity to Paclitaxel (PTX) treatment, respectively (Bargiela-Iparraguirre et al., 2016).

Cisplatin forms upon binding DNA adducts, which lead to cell death by apoptosis (Ho et al., 2016). CDDP stimulates the intrinsic apoptotic pathway controlled by the BCL-2 protein family (Basu and Krishnamurthy, 2010; Maier et al., 2016). This family includes: the anti-apoptotic subfamily, (Bcl-2, Bcl-XL, Bcl-w, Mcl-1, BFL1/A-1, and Bcl-B proteins), the pro-apoptotic subfamily (BAK and BAX), and the BH3-only protein subfamily (BIM, BID, BIK, BAD, BMF, HRK, PUMA, and NOXA proteins) (Delbridge et al., 2016). Under stress, the relative expression of pro- and anti-apoptotic Bcl-2 proteins is modified (Delbridge and Strasser, 2015). BH3-only Bcl-2 proteins are activated either transcriptionally or post-transcriptionally leading to the initiation of apoptosis. DNA damage and growth factor withdrawal target Mcl-1, which will in turn be degraded by the ubiquitin-proteasome system and favor apoptosis induction (Li et al., 2016). Several studies have demonstrated that the overexpression of Bcl-2 is associated with resistance to cytotoxic chemotherapeutic agents in patients with GC (Nakata et al., 1998; Zhuang et al., 2015). The pro-apoptotic protein BAX has been demonstrated to predict clinical responsiveness to chemotherapy in patients with GC (Pietrantonio et al., 2012). Other Bcl-2 family members (Bcl-XL, BAK, and Mcl-1) also have a role in the regulation of chemotherapy-induced apoptosis (Kubo et al., 2016; Matsumoto et al., 2016). This indicates that proteins from the Bcl-2 family play a pivotal role in the determination of cell fate following chemotherapy, through interactions among its members.

Nucleotide excision repair (NER) is the main pathway responsible for the removal of bulky lesions induced by CDDP. The *Xeroderma Pigmentosum* (XP) complementation group of proteins XPA–XPG is involved in NER processes including damage recognition, unwinding, excision, and refilling of DNA (Spivak, 2015). Of particular importance for NER are the two helicases subunits XPB and XPD, which are known to open the DNA helix around the lesion. Then ERCC1 and XPG are recruited and cleave a fragment of the damaged strand. The final step is to fill in the gap thanks to a DNA polymerase and a ligase. The overexpression of some of the components of NER such as ERCC1 has been directly related to increased resistance to CDDP in testicular cancer (Usanova et al., 2010). DSBs are repaired mainly through two pathways, non-homologous end joining (NHEJ) and homologous recombination (HR). BRCA1, a tumor suppressor protein involved in several cancers such as breast cancer, plays a pivotal role in the choice between NHEJ or HR.

During the development of cancer, tumor cells acquire different characteristics and therefore, this intrinsic heterogeneity of the tumor hinders prediction of drug response. We have previously described CHK1 as a biomarker of response to radiotherapy in GC (Bargiela-Iparraguirre et al., 2016). In this manuscript we have compared the process of apoptosis induction in two gastric adenocarcinoma cell lines (AGS and MKN45), which show different sensitivity to CDDP. Our data strongly suggest that MKN45 cells are highly sensitive to CDDP when compared to AGS cells. When we studied the DNA damage repair pathway NER in this cell line, our results suggested that NER activity is impaired in MKN45 cells due to the absence of nuclear translocation of two key NER proteins (XPA and XPD) and the lack of XPC expression. Altogether, these results propose new potential targets that could be used as biomarkers to predict the response to drugs used in the clinical setting.

## Materials and Methods

### Cell Lines

AGS and MKN45 human gastric adenocarcinoma cell lines were acquired from ATCC and cultured in F12-Kings and RPMI medium, respectively (Gibco), and supplemented with FBS 10%. Cultures were maintained at 37°C, 5% CO_2_ and 95% humidity. AGS and MKN45 cells are wild type for TP53 (Bargiela-Iparraguirre et al., 2016). Cell lines were authenticated by genetic profiling using polymorphic short tandem repeat (STRs) loci [System StemElite ID (Promega)]. The analyzed STRs were D21S11, TH01, TPOX, vWA, Amelogenine, CSF1PO, D16S539, D7S820, D13S317, and D5S818. Mycoplasma contamination is routinely tested in our laboratory.

### Chemicals and Plasmid Vectors

Cisplatin was kindly donated from Ferrer FARMA. DAPI was purchased from Sigma-Aldrich. ATR and ATR-DN expression plasmids were kindly donated by Dr. P. Muñoz-Cánoves (Vidal et al., 2005). Crystal violet was purchased from Promega.

### Cell Viability

Viability was determined using a crystal violet-based staining method. Briefly, 5 × 10^4^ cells per well were seeded in 1 ml of completed medium in 24 multiwell dishes, treated with various amounts of CDDP solved in sterile water for 72 h and fixed with 1% glutaraldehyde. After they were washed in 1× PBS, cells were stained with 0.1% crystal violet. A colorimetric assay using 595 nm Elisa was used to estimate the number of cells per well. IC50 were calculated by using the GraphPad Prism program. We used non-linear regression to fit the data to the log (inhibitor) vs. response (variable slope) curve.

### Cell Cycle Analysis

Cells were seeded in 2 ml of completed medium in p60 plates (1.5 × 10^6^ cells per plate) and after appropriate treatment, adherent and non-adherent cells were harvested and fixed overnight in 70% ethanol in phosphate-buffered saline (PBS). For DNA content analysis, the cells were centrifuged and resuspended in PBS containing 1 μg/ml RNase (Qiagen Ltd., Crawley, United Kingdom) and 25 μg/ml propidium iodide, incubated at room temperature for 30 min, and finally analyzed using a Becton Dickinson Flow Cytometer (Cowley, United Kingdom). Data were plotted using Cell Quest Pro software, with 10,000 events analyzed per sample.

### Senescence Assay

A total of 15 × 10^3^ cells were seeded on MW-6 plates in 2 ml of completed medium. Cells were stimulated with CDDP 2.5 μg/ml during 72 h, refreshed with fresh medium and 3 days later β-galactosidase (SA-β-Gal) activity was quantified using the Senescence detection kit (BioVision^1^). Ten areas were counted with the objective 20× in a microscope Nikon Eclipse TS100 and ANOVA1 was performed with IBM SPSS 22 software.

### Western Blotting

Total protein extracts (WCE) were obtained using the previously described lysis buffer (Sanchez-Perez et al., 1998). Nuclear and cytoplasmic cell fractions were obtained as follow: cells were washed with PBS 1×, centrifuged at 3000 rpm for 4 min at 4°C and supernatant was discarded. The pellet was resuspended in cold RBS (20 mM Tris–HCl, pH 7.5, 10 mM NaCl, 3 mM MgCl_2_, containing protease, and phosphatase inhibitors) for 10 min and 10% NP40 for 20 min. Next was centrifuged at 3000 rpm for 4 min at 4°C, and the supernatant, which contained the cytoplasmic proteins, was collected. The pellet was resuspended with cold RBS and 10% NP40, centrifuged at 3000 rpm for 4 min at 4°C, washed twice more with cold RBS, centrifuging between each wash and discarding the supernatant in each case. The pellet was resuspended in Nuclear Lysis Buffer (20 mM Tris–HCl, pH 7.5, 0.4 M NaCl, 1 mM EDTA, containing protease, and phosphatase inhibitors) for 15 min and sonicated for 12 min. Finally, it was centrifuged at 13200 rpm for 5 min at 4°C, and the supernatant, which contained nuclear proteins, was collected. Twenty micrograms of WCE and 30 μg of nuclear and cytoplasmic fractions per sample were loaded in 15% (for Bcl2-family), 10% (MAPKs, XPA, and ERCC1), or 8% (PARP-1, XPC, and XPD), respectively, SDS-PAGE polyacrylamide gels, and then transferred onto nitrocellulose membranes, followed by immunodetection using appropriate antibodies. Antibodies against the following proteins were: PARP-1 (sc-7150), Mcl-1 (sc-819), DUSP1 (sc-370), JNK (sc-827), p38 (sc-535), ERK1/2 (sc-154), p-cJun (sc-822), p-ATF2 (sc-135686), XPA (1:500, sc-28353), XPC (sc-74411), ERCC1 (sc-10154) and were purchased from Santa Cruz Technology. The following antibodies were purchased from Cell Signaling Technology: Cleaved Caspase-3 (Asp175) (#9661), Bad, Bax, BID, Bak, Bcl-XL (#9942), p62 (#5114), p-p38 (1:2000, #4631), p-ERK1/2 (1:2000, #9106), and XPD (#4636). The polyclonal antibody for phosphorylated JNK, Rabbit, (pTPpY) was acquired from Promega Corporation-Spain. Finally, the FLAG (1:2000) and α-tubulin (1:10000) antibodies were purchased from Sigma-Aldrich. Unless otherwise specified, all the above antibodies were used at a working dilution of 1:1000.

### RT-PCR

AGS and MKN45 cells were seeded in p60 plates (1.5 × 10^6^ cells per plate in 2 ml of completed medium) and treated with 10 μg/ml CDDP for different times (0–24 h) and RNA was isolated as previously described. The relative mRNA level was calculated by the *C*t (2^−ΔΔ^*^C^*^t^) method, using GAPDH as an endogenous reference and HaCat cells (human normal keratinocytes cells) as control cells. ΔΔCT represents the difference between the mean ΔCT value of the cells tested and the mean ΔCT value of the calibrator, both calculated after the same PCR run, in which ΔCT is the difference between the CT of the target mRNA and the CT of the endogenous reference (GAPDH) of the same sample. The relative quantitative value was expressed as 2^−ΔΔ^*^C^*^t^. Primer sequences used were previously described (Chen et al., 2016).

### Immunofluorescence

Cells were fixed in formaldehyde for 20 min, washed with PBS and permeabilized with Triton 0.5% for 10 min, blocked with BSA 5% for 1 h. Samples were incubated overnight with the primary antibody at 4°C, followed by a 1-h incubation with the adequate secondary antibody at room temperature. DNA was stained with DAPI. Fluorescence microscopy was performed using a NIKON Eclipse 90i, and for the image analysis the software program Nikon NIS-Elements and Image J were used. The primary antibodies used in our study were Monoclonal antibody for CDDP-induced Pt(GG) intrastrand adducts in DNA. Cod: R-C18 from ONCOLYZE; γ-H2AX^Ser139^, purchased from Millipore, and XPD (1:100; #1284) from Sigma-Aldrich. All secondary antibodies, conjugated with Alexa Fluor 488, (1:500) were purchased from Invitrogen. γ-H2AX foci were quantified with Cell Profiler software and analyzed with IBM SPSS 22 with two-way ANOVA test.

### Comet Assay

We performed the comet assay under alkaline conditions as described by Landi et al. (1998) and Singh et al. (1988). Briefly, cells were treated for 3 h with CDDP (10 μg/ml), embedded in agarose and keep on ice. Before electrophoresis performance, we introduced single strand breaks by using 2 Gy IR, and immediately immersed in lysis buffer. This step is done since the damage induced by CDDP cannot be resolved in alkaline conditions that allow the electrophoretic migration of the DNA fragments according to their content in adducts. For the repair assay, cells were then allowed to recover from the induced damage thorough washes in PBS and incubation at 37°C in 5% fresh media for 30 min, 1, 2, and 3 h. TM was calculated with ImageJ OpenComet plugin, data were analyzed with IBM SPSS 22 with Kruskal–Wallis and Mann–Whitney tests.

### Scoring DNA Damage

Immediately before imaging analysis, slides were stained with 60 μl of a 1 μg/mL ethidium bromide solution for 10 min and covered with coverslips before imaging using a 20× objective under a fluorescence microscope (NIKON90i). Experiments were performed in duplicate. One hundred consecutive cells (50 from each duplicate slide) were randomly selected (carefully avoiding the borders of the slides), scored and quantified by ImageJ analysis software. The extent of the damage was measured quantitatively by the tail moment (TM), defined as the product of the percentage of DNA in the comet tail and the tail length (Gutierrez-Gonzalez et al., 2013). For the analysis of repair kinetics, the percentage of residual DNA damage (% RD) at time *t* after CDDP was calculated as follows: % RD = 100 × [(DNA damage at time *t* after CDDP-DNA damage in control cells before CDDP)/(DNA damage immediately after CDDP-DNA damage in control cells before CDDP)] (Marcon et al., 2003).

### Cellular Uptake and Distribution by ICP-MS

To assess the uptake of cisplatin and its intracellular distribution, AGS and MKN45 cells were seeded in p60 plates in 2 ml of completed medium (1.5 × 10^6^ cells per plate) and were exposed to the drug and CDDP content in the nucleus and cytoplasm was analyzed by ICP-MS. The cells were seed into p60 tissue culture plates at a density of 1.5 × 10^6^ cells, and allowed to attach overnight at 37°C. The adherent cells were incubated with CDDP 10 μg/ml for 3 h. Then, the cells were washed with PBS and detached from plates with trypsin. In suspension, the cells were counted, 10^6^ cells were separated, washed twice with cold PBS, and centrifuged (1000 rpm, 5 min) to obtain a pellet. In order to disrupt the cellular membrane, the pellet was resuspended in lysis buffer (10 mM Tris, 1.5 mM MgCl_2_, 140 mM NaCl, pH 8.0–8.3) with Nonidet P40 (0.02%). After 15 min incubation on ice, the suspension was centrifuged at 1300 *g* for 2 min at 4°C and nuclear fraction (pellet) separated from the cytoplasmic fraction (supernatant). The Pt content in the two fractions was measured, after digestion in open vase with ultrapure HNO_3_ (65%), H_2_O_2_, and HCl, evaporated and resuspended in ultrapure water to obtain a 2.0% (v/v) nitric acid solution, by ICP-MS on a ICP-MS NexION 300xx PerkinElmer instrument, with ^187^Rhenium used as internal standard. The protocol was performed at the Elemental analysis unit of UAM which fulfill the Quality Management System IQNet – ISO 9001:2008.

### Statistical Analysis

The Kruskal–Wallis test, a non-parametric procedure was used to compare responses, as measured by TMs. Repair ability was measured as the percentage of residual damage at time *t* which was calculated as described above. At least 100 nuclei were measured per condition in IF assays. All data were presented as mean ± standard deviation (SD) after three independent experiments. Statistical significance (at *p* < 0.05) was determined using Student’s *t*-test and one-way or two-way ANOVA, using IBM SPSS 22 software.

## Results

### Cell Death After Cisplatin Treatment in Gastric Cancer Cell Lines

**FIGURE 1:**
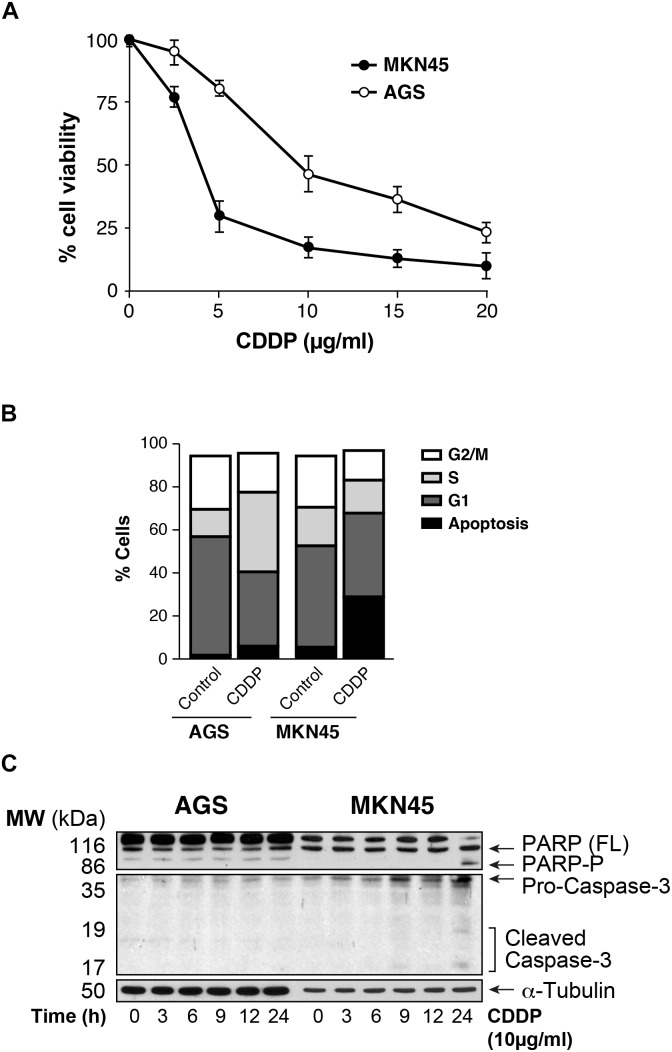
AGS and MKN45 show different sensitivity to CDDP. **(A)** Cell survival percentage of AGS and MKN45 cells after 72 h of CDDP treatment. Cells were treated with increasing concentrations of CDDP (0–20 μg/ml). The percentage of viable cells was quantified by the crystal violet method. Data represent the mean values obtained in three experiments performed in quadruplicate. **(B)** Cell cycle profile of AGS and MKN45 cells after 24 h of CDDP treatment (10 μg/ml). DNA content was assessed by flow cytometry, and cell cycle distribution was analyzed using Cell Quest Pro software. The graph shows the percentage of each cell cycle, which is given as the mean ± standard deviation of three experiments. G2/M, cells in G2 or Mitosis; S, cells in phase of synthesis of DNA; G1, cells in G1. **(C)** Cleavage of PARP-1 and Caspase-3 activation were detected by western blot (WB) in cells harvested at the indicated times after CDDP treatment (10 μg/ml). α-Tubulin was used as loading control. WB images are representative of the results obtained in three different experiments made in the same conditions.

We analyzed the viability of AGS and MKN45 cell lines after treatment with CDDP. Our results showed that survival rate decreases in a dose dependent manner after CDDP exposure in both cell lines, being AGS more resistant than MKN45 cells (IC_50_ 7.6 μg/ml vs. IC_50_ 2 μg/ml, respectively) (**Figure 1A**), and at different times of exposure (**Supplementary Figure 1**). Then we studied the cell cycle profile and the results showed that the percentage of apoptotic AGS cells is 6.34%, in contrast to the 28.38% of apoptotic MKN45 cells (**Figure 1B**). Based on these data, we looked at specific biochemical markers such as caspase-3 activation and PARP cleavage after equitoxic CDDP treatment. The western blot analysis of MKN45 cells showed PARP and caspase-3 cleavage, indicating proteolysis and activation, respectively. In contrast, CDDP fails to activate caspase-3 and PARP proteolysis in AGS cells (**Figure 1C**), and this result suggests a different apoptosis induction in both cell lines. Rationally, we checked out additional pathways that could be involved in cell death, such us senescence and autophagy. In order to study senescence, we analyzed SA-β-galactosidase activity in AGS and MKN45 cells treated with CDDP (**Supplementary Figure 2A**). Our results showed similar induction of senescence in both cell lines. To analyze autophagy, we use western blot analysis of p62 (one of the most significant markers in autophagy). The p62 expression in GC cells is similar in both cell lines revealing p62 degradation (**Supplementary Figure 2B**). The different viability caused by CDDP in AGS and MKN45 cells could be produce by a different apoptosis activation.

### Intrinsic Apoptosis Pathway Mediated by Mcl-1 in MKN45 Cells

**FIGURE 2:**
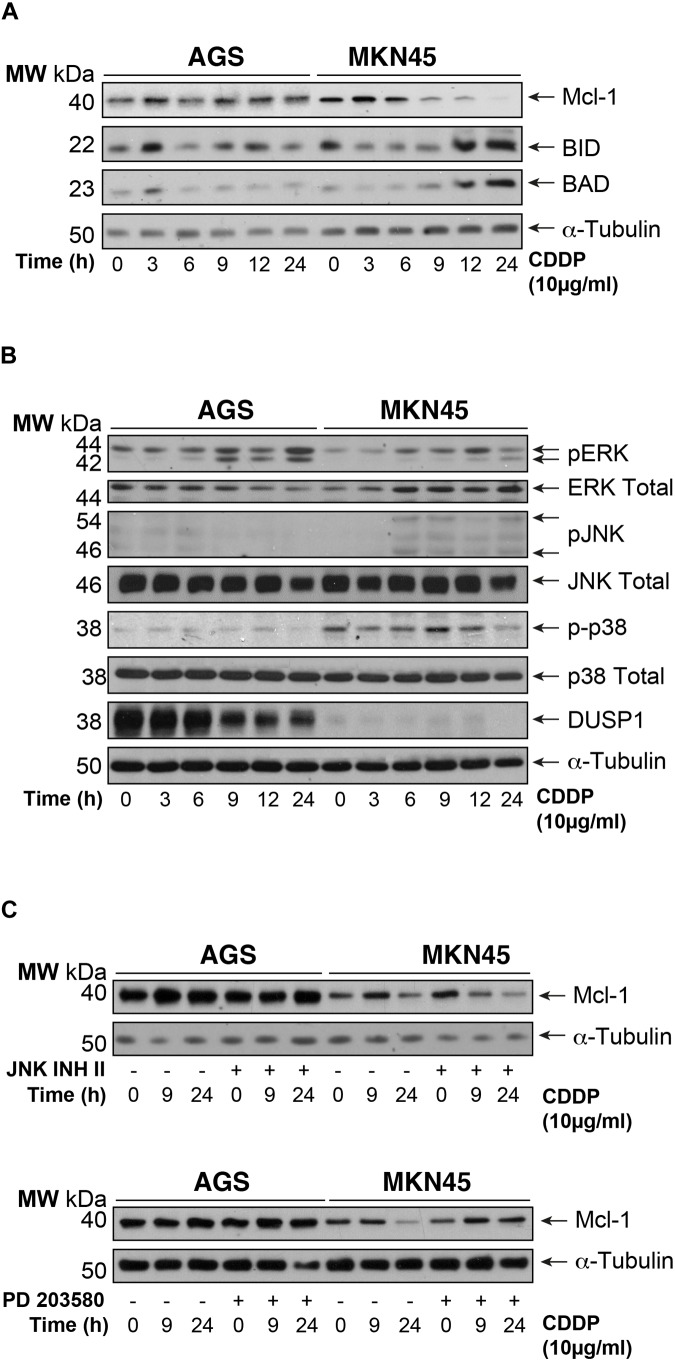
Intrinsic apoptosis pathway by CDDP is mediated by Mcl-1 in MKN45 cells. **(A)** Bcl-2 protein family members were detected by western blot in AGS and MKN45 cells treated with CDDP (10 μg/ml) at the indicated periods of time. Mcl-1, Bid, and Bad were detected by using specific antibodies. **(B)** Cells were treated as in panel **(A)**. ERK, JNK, p38 (phosphorylated and total forms), and DUSP1 expression were detected by using specific antibodies. **(C)** AGS and MKN45 cells were pretreated during 30 min with 10 μM JNK Inhibitor II or p38 inhibitor (SB203580) before CDDP treatment. Mcl-1 was detected by western blot at the indicated times after treatment. α-Tubulin was used as loading control. WB images are representative of the results obtained in three different experiments made in the same conditions.

To gain insight into the mechanism of apoptosis induction of CDDP, we analyzed the expression of the pro-survival BCL2-like proteins (Bcl-XL and Mcl-1), the pro-apoptotic factors and BH3-only members: Bak, Bax, Bid, and Bad. We observed downregulation of Mcl1 expression in MKN45 cells (almost 70%) but not in AGS cells after 9 h of exposure to CDDP (**Figure 2A**). By contrast, the expression of the pro-apoptotic proteins Bid and Bad strongly increases from 9 to 24 h after treatment in MKN45 cells, whilst no effect was observed in AGS cells. No differences were found in the other Bcl-2 member studied (**Supplementary Figure 2C**).

Mcl-1 degradation is controlled by JNK and p38 MAPKs thus, we analyzed their activation. Surprisingly, CDDP slightly activated the stress kinases JNK and p38 only in MKN45 cells but none in AGS cells (**Figure 2B**). In addition, we analyzed the DUSP1 levels, the main phosphatase responsible of inactivation of the three MAPKs. Our data indicated overexpression of DUSP1 in AGS cells, which clearly explains the lack of JNK or p38 activation in this type of cell line (**Figure 2B**). We observed the phosphorylation of ERK kinases equally in both cell lines, typically produce by cisplatin. To verify the role of p38 and JNK activity on Mcl-1, we used the pharmacological inhibitors SB203580 and JNK Inhibitor II for p38 and JNK, respectively. As a control for the activity of these inhibitors, we analyzed the phosphorylation of specific targets for these kinases, such as ATF2 and c-Jun (**Supplementary Figure 2D**). Our results indicated that p38 inhibition blocked Mcl-1 degradation in MKN45 cells (**Figure 2C**), but not by JNK inhibition.

These results strongly suggest that CDDP induces apoptosis in MKN45 cells due to Mcl-1 degradation, in a process that depends on p38 activity and upon the upregulation of Bid and Bad.

### DNA Damage Repair and Impair in Gastric Cancer Cells Treated With CDDP

**FIGURE 3:**
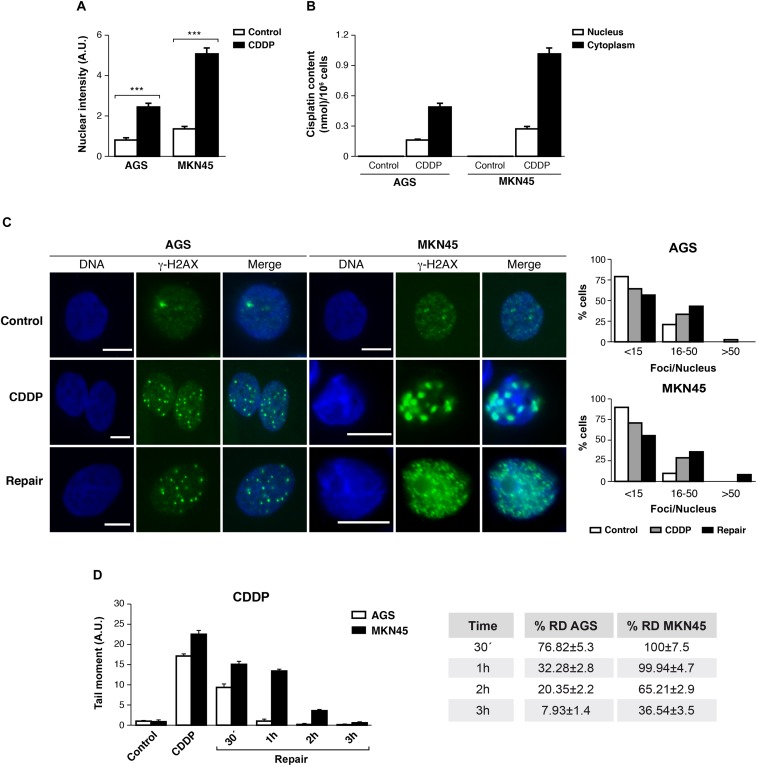
DNA repair is impaired in MKN45 cells. **(A)** Graph represents nuclear fluorescent intensity of DNA adducts formed after treating AGS and MKN45 with CDDP (10 μg/ml). Control cells (white bars) were untreated, or treated CDDP (black bars) for 3 h. DNA adducts were detected by using anti-(Pt-DNA) antibody. **(B)** Subcellular distribution of CDDP in GC cells after 3 h of 10 μg/ml treatment. White bars represent the CDDP content in nucleus and black bars in cytoplasm. Data are expressed as the mean values of the metal (nmol cisplatin) content in the nucleus or cytoplasm per million cells obtained in three different experiments. **(C)** AGS and MKN45 cells were treated as in panel **(A)**, and they were allowed to recover in normal media for 1 h before harvesting. γ-H2AX foci were detected by Immunofluorescence, using DAPI to stain nuclear DNA. Graphs represent the percentage of nucleus within less than 15, between 15 and 50 and more than 50 γ-H2AX/nuclei for each condition. **(D)** Graphs represent the mean tail moment (TM) measured in at least 50 cells per duplicated in each condition after CDDP treatment (10 μg/ml). Tables present the percentage of residual damage (RD) after the repair period. ^∗∗∗^*p* < 0.001.

The ability of CDDP to reach and react with the DNA is ascribed to its anticancer activity. We tested then, the amount of CDDP-adducts formed in AGS and MKN45 quantifying the fluorescence intensity of the nuclei by using a specific antibody, which recognizes CDDP-adducts. In both cell lines, we found the nuclear intensity increased after 3 h of CDDP treatment compared with the control (**Figure 3A** and **Supplementary Figure 3**). In order to get more evidences from the damage caused by cisplatin in our previous experiment; we measured the platinum content into the nucleus and cytoplasm by ICP-mass spectrometry. AGS and MKN45 cells were incubated with cisplatin for 3 h and the nuclear fraction was separated from the cytoplasmic fraction, and measured by ICP-MS (**Figure 3B**). The platinum content in both fractions is much lower in the AGS cell line than in the MKN45. Within these values, it is worth noticing that, in both cell lines, the platinum content is almost as high in the nucleus as in the cytoplasm, which also correlates with the early time of measurement (3 h). Overall, these values proved that the damage detected in the fluorescence experiment is caused by the platinum binding.

We also analyzed the double-strands breaks generated by CDDP in these cells, quantifying the number of DNA damage foci per cell using antibodies against γ-H2AX^Ser139^. Our data showed that the number of foci per cell increased significantly 3 h after CDDP treatment, on a range of 16 to 50 foci per nuclei. The DNA damage foci numbers, after withdrawal of cisplatin, decreased in AGS (range > 50 foci/nuclei) and kept increasing in MKN45 cells (**Figure 3C**).

Next, we performed a comet assay to evaluate the repair rate in these cells after CDDP treatment. Tail moment data showed that AGS cells were able to repair CDDP adducts within 1 h; by contrast, MKN45 cells needed at least 3 h to remove the damage. Accordingly, the percentage of Residual Damage (% RD) reached 7.93 ± 1.4 in AGS versus 36.54 ± 3.5 in MKN45 cells after 3 h of repair (**Figure 3D**). These results strongly suggest that AGS cells repair CDDP induced DNA lesions more efficiently than MKN45 cells.

### Nuclear Excision Repair Proteins Expression After CDDP Treatment in AGS and MKN45 Cells

**FIGURE 4:**
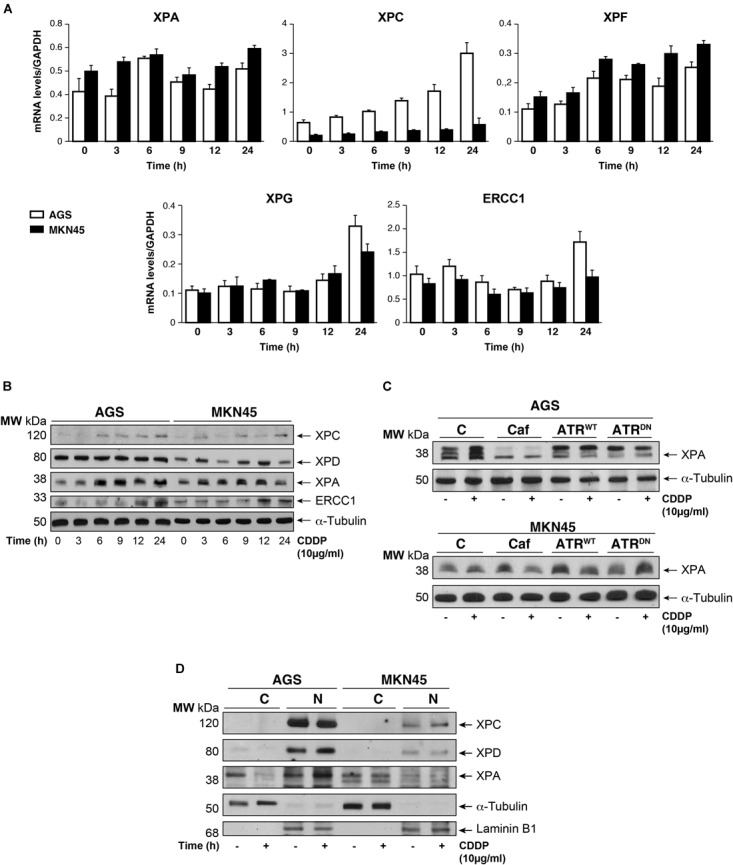
CDDP selectively increased NER proteins in GC cells. **(A)** AGS and MKN45 cells were treated with 10 μg/ml CDDP and harvested at the indicated times. RT-qPCR was used to quantify the mRNA levels of XPA, XPC, XPF, XPG, and ERCC1 by using specific primers (see section “Materials and Methods”). The graphs represent the relative levels of each gene using ΔΔCT referred to the levels on a control no tumorigenic cell line HACAT, and using GAPDH as endogenous control. **(B)** XPC, XPD, XPA, and ERCC1 protein levels were detected by western blot in AGS and MKN45 cells after CDDP (10 μg/ml) treatment at the indicated times. α-Tubulin was used as a loading control. **(C)** XPA levels in AGS and MKN45 cells after 6 h of CDDP treatment (10 μg/ml) in the presence of caffeine (80 mM) and transfected with ATR^WT^ or ATR^DN^ plasmids. Arrow in XPA band indicates the non-processed protein. **(D)** Nuclear and cytoplasmic localization of XPC, XPD, and XPA proteins were detected by western blot in AGS and MKN45 cells after 6 h of CDDP treatment (10 μg/ml) and cellular fractioning (C, Cytoplasm; N, Nuclei). α-Tubulin was used as cytoplasmic protein loading control and lamin B1 was used as nuclear protein control. The experiments were repeated three times with similar results.

The damage produced by cisplatin in the GC cells is generally repair by NER system. We analyzed this pathway quantifying the mRNA levels of different NER factors by RT-qPCR (**Figure 4A**). Our results showed that the expression of the XPA and the XPF is equally induced in both cell lines, in addition, no major differences were observed in basal levels of the mRNAs. Moreover, XPC highly increases after cisplatin treatment in AGS, but this effect is not shown in MKN45. Lastly, XPG and ERCC1 stayed the same with a slight increase at 24 h. (**Figure 4A**). We also quantify a homologous recombination repair (HRR) system, with no additional changes and the data from BRCA1 and Rad51 have been collected in **Supplementary Figure 4**. Next, we verified this NER system pathway by WB analysis of the protein expression levels. We observed a clear induction of XPC and ERCC1 expression (**Figure 4B**) from 12 to 24 h of treatment in both cell lines being slightly lower in MKN45. Interestingly, we observed a modified pattern of the XPA expression (**Figure 4B**) showing some extra-bands indicative of post-transductional modifications (**Figure 4C**). ATR is known to increase NER activity by phosphorylating and stabilizing XPA in response to DNA damage (Lee et al., 2014). In order to determine the effect of ATR or ATM kinases over XPA, we treated MKN45 cells with caffeine in the presence and in the absence of CDDP. Our results showed that pretreatment with caffeine reduce the mobility shift of XPA, which points to changes in the phosphorylated state of this protein (**Figure 4C**). To verify such phosphorylation, AGS and MKN45 cells were transfected with ATR^WT^ or ATR^DN^ expression vectors, and consistently with our previous data, the shift of XPA in response to CDDP decreased by expressing ATR dominant negative plasmid (**Figure 4C**). With these data in hand, we can tell that DNA repair factors are controlled differently between both cell lines in response to CDDP.

We even gain more insight about the repair mechanism examining XPA, XPC, and XPD localization. Subcellular fractionation and WB analysis revealed that XPC in Nucleus was highly expressed in AGS cells (**Figure 4D**); however, the amount of XPC in MKN45 cells was considerably reduced, following our previous results with RT-qPCR and WB. Although XPD is restricted in the nucleus in both cell lines, we observed after cisplatin treatment a low XPD increase in AGS cells and none in MKN45. Finally, we observed that XPA, the key rate-limiting factor for NER (Kang et al., 2011), was translocated from the cytosol into the nucleus after DNA damage induced by CDDP in AGS cells. Interestingly, we did not find relocalization of XPA in MKN45 cells where this factor remains localized in the cytoplasm after CDDP treatment (**Figure 4D**).

## Discussion

In this manuscript, we seek to predict CDDP response in GC cells. It is known that CDDP-induces apoptosis by a general pathway in normal cells, however, cancer cells show specific particularities leading, in some cases, to avoid the cell death. CDDP binds to the DNA and induces bulky lesions which are mainly repaired by NER mechanism and if the cells are not able to repair the damage they end dying by apoptosis induction (Florea and Busselberg, 2011). Resistance mechanisms to CDDP avoids or decreases the efficiency to treatment, so some efforts have been done in order to find combined therapy using target pathways related with resistance (Galluzzi et al., 2012).

We described here, that CDDP induces apoptotic cell death in CDDP-sensitive GC cells (MKN45), through both downregulation of the anti-apoptotic protein Mcl-1, and induction of the pro-apoptotic BH3-only proteins Bad and Bid. Our findings are in agreement with recent reports revealing that suppression of the FoxM1/Mcl-1 pathway impairs cell viability and thus increases sensitivity to CDDP in GC cells (Li et al., 2016). Mcl-1 promotes cell transformation, cancer survival, and resistance to chemotherapy, and selective Mcl-1 inhibitors competitively engage its binding groove, mimicking the structural mechanism of action of native sensitizer BH3-only proteins (Rezaei Araghi et al., 2018). Our data suggest that CDDP mainly uses the Bad-dependent apoptotic pathway in GC. Moreover, those markers are essential for caspase 9/3 activation that finally produces cell death. And this Apoptotic induction is observed as a consequence of cisplatin response in MKN45 and it is not observed in AGS. These data reinforce the need to identify specific Bcl-2-family proteins as targets for treatment improvement. The development of compounds mimicking the function of BH3-only proteins is an emerging area of research. Thus, many inhibitors had been developed in the last decade, together with clinical trials (still ongoing) to study their effects in combination with chemotherapy (Delbridge et al., 2016).

Chemoresistance is a major problem that leads to treatment failure and death in GC patients. Among the various chemoresistance mechanisms, overexpression of drug-resistance proteins including drug transporter and DNA repairing systems, are important defense mechanisms of cancer cells against chemotherapeutic drugs. We have found that the less amount of CDDP in the nucleus in resistant cells than in sensitive cells, and the amount directly correlates with DNA damage amount. One possible mechanism is that sensitive cells have overexpression of proteins involved in the CDDP transport, but our preliminary results do not confirm this hypothesis (data not shown). Drug efflux transporters, including P-gp (MDR1) and MRP1, are capable of reducing the intracellular drug concentration. The resistance to 5-FU and irinotecan due to the MRP associated cellular efflux processes has also been reported (Suzuki et al., 2001).

The other mechanism must be related to DNA repair mechanism. Our results point to this fact, as AGS cells showed a competent DNA repair but MKN45 cells showed an inefficient DNA repair. The NER system involves more than 30 protein–protein interactions and removes DNA adducts caused by platinum-based chemotherapy (Jung and Lippard, 2007). XPC is a DNA-damage-recognition gene active at the early stage of DNA repair. In the present study, we study the expression levels of genes involved in NER in GC cancer cells. This analysis revealed that expression of XPC mRNA after CDDP treatment significantly increased in AGS cells compared with MKN45 meaning that it may be a potential target for chemotherapy of GC. This observation is in agreement data reported for CRC cells (Zhang et al., 2018). XPD and XPA are important proteins (helicase and recognition, respectively) within the NER pathway. Our results show that the expression and translocation of XPD and XPA to the nucleus in MKN45 cells is impaired. The importance of this result must be related with ERCC1 which is overexpressed in cancer cells. The XPA–ERCC1 complex seems to be one of the most promising targets in this pathway. In fact, the only known cellular function for XPA is to recruit ERCC1 to the damaged point. These results open up two new lines of research: (a) for the discovery of new NER inhibitors aimed at improving the efficacy of current platinum-based therapy by modulating the XPA–ERCC1 interaction (Gentile et al., 2016) and (b) for the mechanism studies of XPA and XPD downregulation in MKN45 cells as a predictor of CDDP efficacy.

**FIGURE 5:**
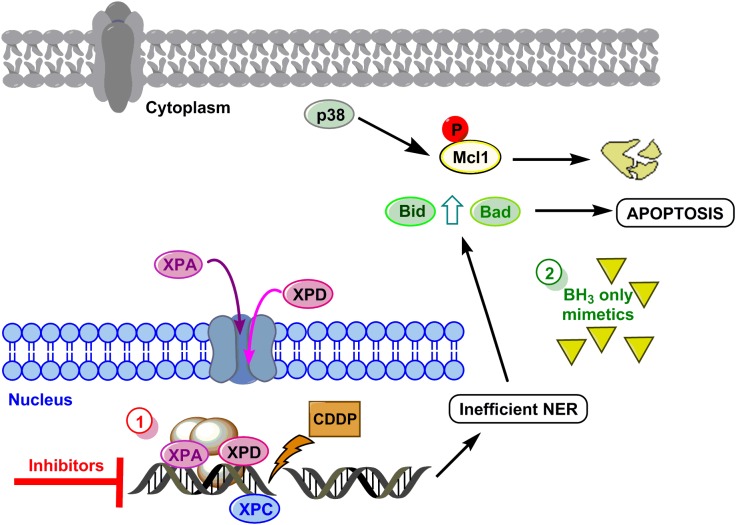
Hypothetical model to sensitize GC. DNA damage caused by CDDP leads to activation of NER, XPC is already in the nucleus and proteins XPA and XPD translocate to the nucleus to repair the lesion. Resistant cells repair properly the DNA and survive, however, if repair is not efficient, the mitochondrial apoptosis pathway is induced by Mcl-1 degradation and Bid and Bad induction. Option 1 (red circle) proposes the inhibition of XP proteins, and option 2 (green circle) proposes the use of BH_3_ only mimetics.

To summarize, we propose a model to sensitize GC cells in those cases showing resistance to the drug based on the data obtained in AGS and MKN cell lines (see **Figure 5** for details). The first option of this model embrace targeting the DNA repair pathways (XPC, XPD, and XPA) surmounting CDDP resistance in GC cells. The second option will be mimicking the BH3-only proteins.

## Author Contributions

IS-P conducted the experimental design. IS-P, RP, and AQ drafted the manuscript. JB-I and NP-L performed the experiments and analyzed the data. GD helped to perform the comet assay. AQ helped to perform the ICP-mass spectrometry. All authors contributed to the interpretation of the data, revised the manuscript critically, and approved the final manuscript.

## Conflict of Interest Statement

The authors declare that the research was conducted in the absence of any commercial or financial relationships that could be construed as a potential conflict of interest.

## Footnotes

1 http://www.biovision.com/

## Abbreviations

CDDP: cisplatin
DAPI: 4’,6-diamidino-2-phenylindole
ERCC1: excision repair cross-complementation group 1
GAPDH: glyceraldehyde 3-phosphate dehydrogenase
GC: gastric cancer
IF: immunofluorescence
NER: nucleotide excision repair
PCR: polymerase chain reaction
RT: reverse transcription
XP-A: -C, -D, -G, -F, xeroderma pigmentosum group A, C, D, G, F.
