# Supplementary figures and images for: XPA, XPC, and XPD Modulate Sensitivity in Gastric Cisplatin Resistance Cancer Cells
Source: Front Pharmacol. 2018 Oct 17;9:1197. doi: 10.3389/fphar.2018.01197 (PMC6199368; doi:10.3389/fphar.2018.01197)

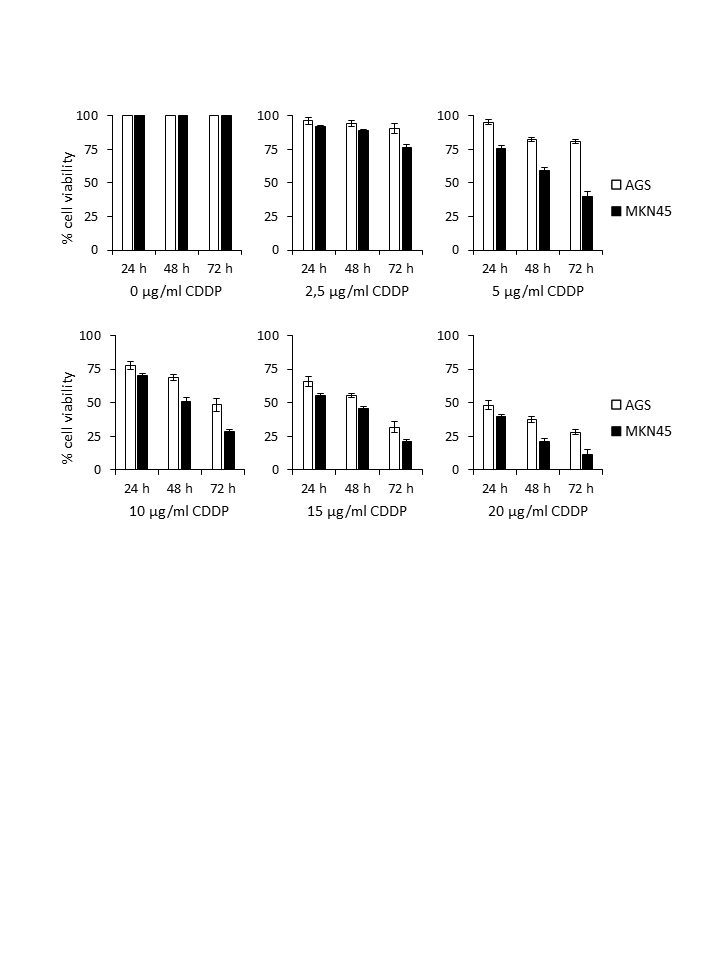

Supplement: Supplementary file 1 [file Image_1.TIF]

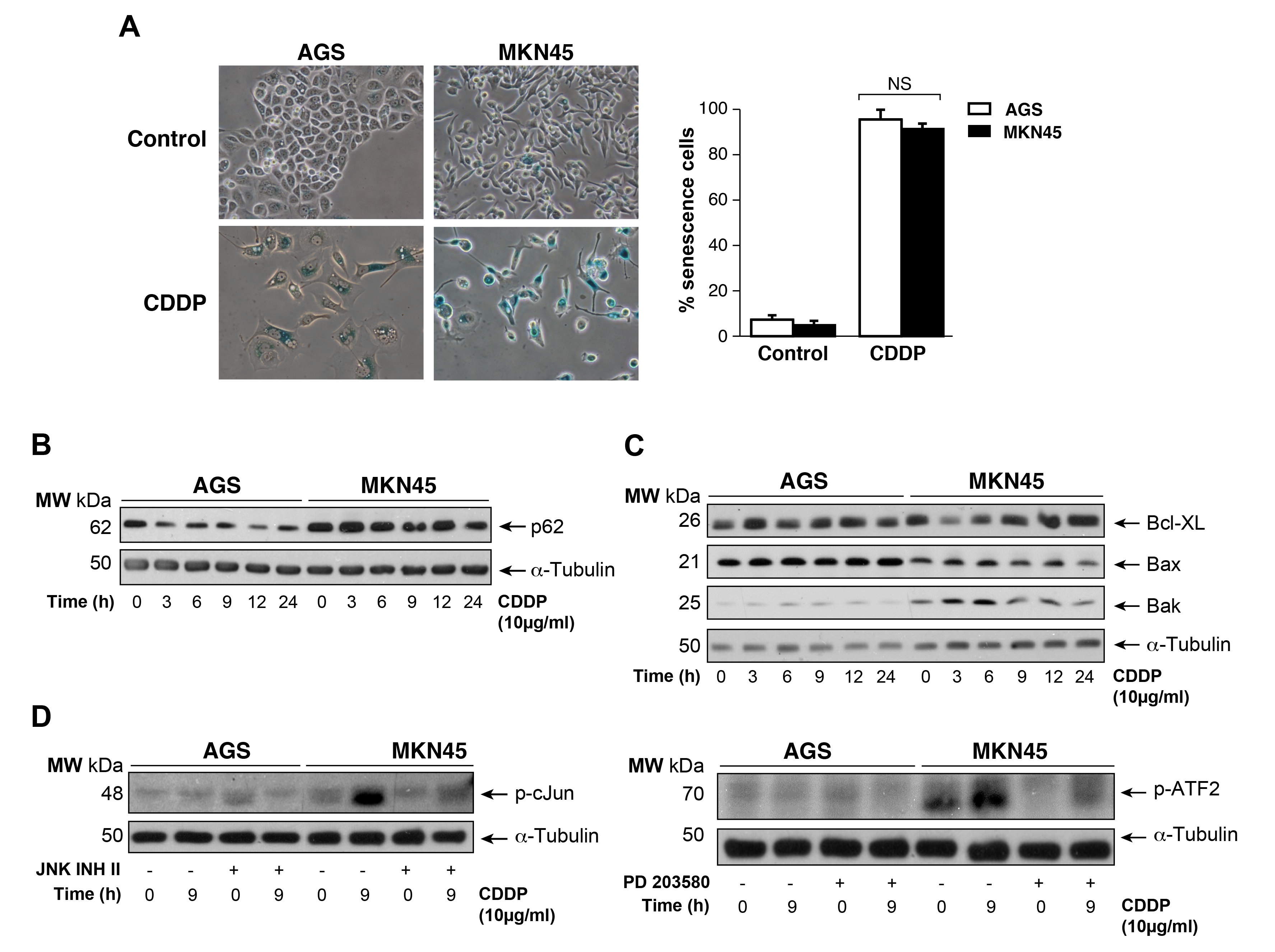

Supplement: Supplementary file 2 [file Image_2.TIF]

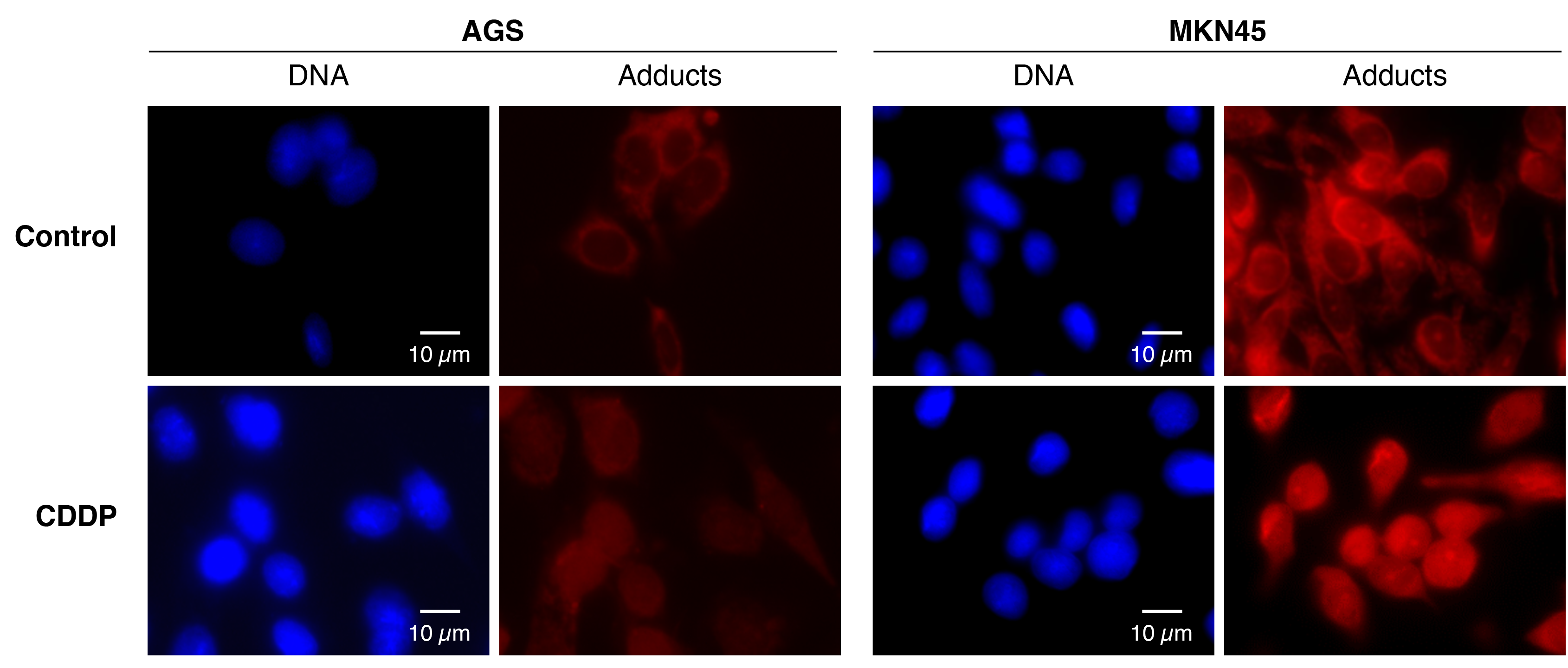

Supplement: Supplementary file 3 [file Image_3.TIF]

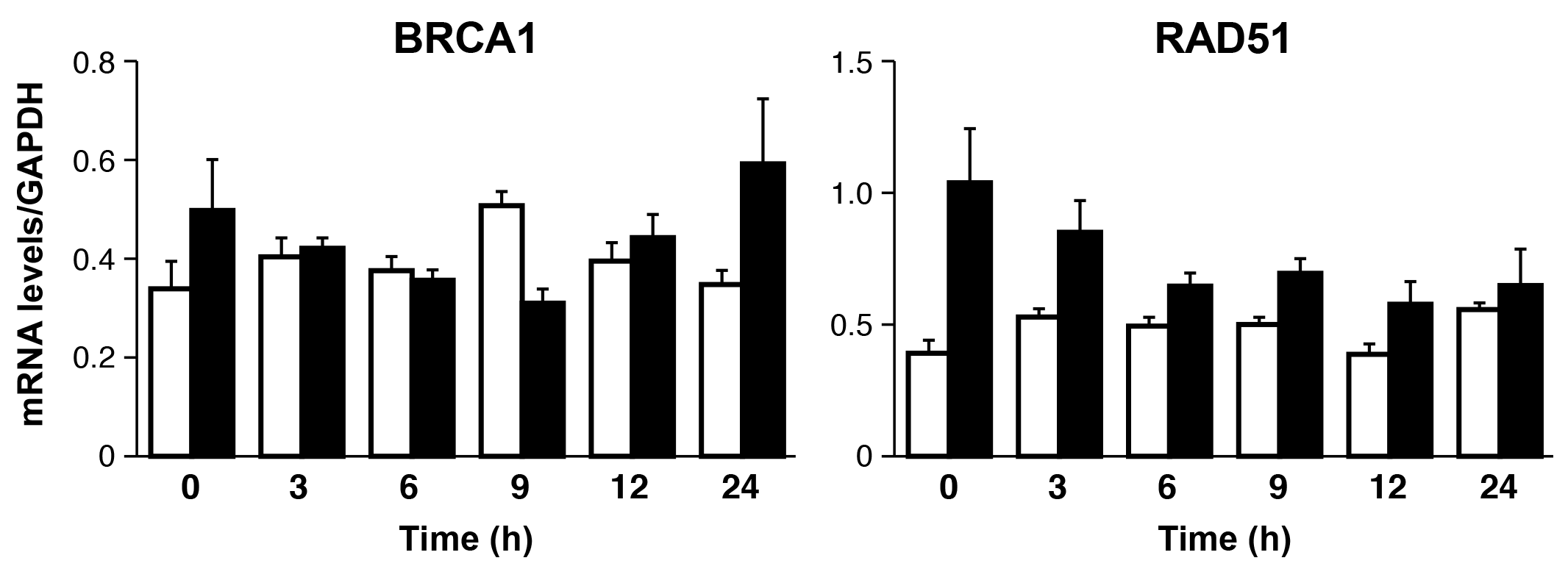

Supplement: Supplementary file 4 [file Image_4.TIF]
