# Supplementary material for: XPA, XPC, and XPD Modulate Sensitivity in Gastric Cisplatin Resistance Cancer Cells
Source: Front Pharmacol. 2018 Oct 17;9:1197. doi: 10.3389/fphar.2018.01197 (PMC6199368; doi:10.3389/fphar.2018.01197)
Supplement: Supplementary file 5 [file Table_1.DOCX]

**Supplementary Figure 1.** A) Cell survival percentage of AGS and MKN45 cells after CDDP (0-20µg/ml) treatment for 24, 48 and 72 hours of exposition. Graph bars represent the percentage of viable cells referred to untreated cells. Data represent the mean values obtained in three experiments performed in quadruplicate.

**Supplementary Figure 2.** A) Representative images of AGS and MKN45 senescent cells after 72h of CDDP (2,5 µg/ml). The graph represents the percentage of senescent cells for each experimental condition. B) AGS and MKN45 cells were treated with CDDP (10 µg/ml) for the indicated periods of time. Immunodetection of p62 protein was carried out by using specific antibodies. C) Bcl-2 protein family members Bcl-XL, Bax and Bak were detected by western blot using specific antibodies in AGS and MKN45 cells treated with CDDP (10 µg/ml) at the indicated periods of time. D) Western blots detecting p-ATF2 and p‐cJUN in AGS and MKN45 cells as control for SB203580 or JNK Inhibitor II activity. α-tubulin was used as a loading control. WB images are representative of the results obtained in three different experiments made in the same conditions.

**Supplementary Figure 3.** Immunofluorescence images showing CDDP adducts formed in the DNA of AGS and MKN45 cells. As opposed to control cells were not treated and CDDP cells were treated for 3h with 10 µg/ml treatment. DNA adducts were detected by using anti-(Pt-DNA) antibody, using DAPI to stain nuclear DNA. Images were taken with a NIKON Eclipse 90i microscope (63x objective).

**Supplementary Figure 4**. AGS and MKN45 cells were treated with 10µg/ml CDDP and harvested at the indicated times. RT-QPCR was used to quantify the mRNA levels of RAD51 and BRCA1 by using specific primers (see M&M). The graphs represent the relative levels of each gene using ∆∆CT referred to the levels on a control no tumorigenic cell line HACAT, and using GAPDH as endogenous control. The experiments were repeated three times with similar results.
